# Antiviral Properties of Lactoferrin—A Natural Immunity Molecule

**DOI:** 10.3390/molecules16086992

**Published:** 2011-08-16

**Authors:** Francesca Berlutti, Fabrizio Pantanella, Tiziana Natalizi, Alessandra Frioni, Rosalba Paesano, Antonella Polimeni, Piera Valenti

**Affiliations:** 1 Department of Public Health and Infectious Diseases, Sapienza University of Rome, Rome 00185, Italy; 2 Department of Woman Health and Territorial Medicine, Sapienza University of Rome, Rome 00185, Italy; 3 Department of Oral Sciences and Maxillofacial Surgery, Sapienza University of Rome, Rome 00185, Italy

**Keywords:** lactoferrin, virus, viral infection

## Abstract

Lactoferrin, a multifunctional iron binding glycoprotein, plays an important role in immune regulation and defence mechanisms against bacteria, fungi and viruses. Lactoferrin’s iron withholding ability is related to inhibition of microbial growth as well as to modulation of motility, aggregation and biofilm formation of pathogenic bacteria. Independently of iron binding capability, lactoferrin interacts with microbial, viral and cell surfaces thus inhibiting microbial and viral adhesion and entry into host cells. Lactoferrin can be considered not only a primary defense factor against mucosal infections, but also a polyvalent regulator which interacts in viral infectious processes. Its antiviral activity, demonstrated against both enveloped and naked viruses, lies in the early phase of infection, thus preventing entry of virus in the host cell. This activity is exerted by binding to heparan sulphate glycosaminoglycan cell receptors, or viral particles or both. Despite the antiviral effect of lactoferrin, widely demonstrated *in vitro* studies, few clinical trials have been carried out and the related mechanism of action is still under debate. The nuclear localization of lactoferrin in different epithelial human cells suggests that lactoferrin exerts its antiviral effect not only in the early phase of surface interaction virus-cell, but also intracellularly. The capability of lactoferrin to exert a potent antiviral activity, through its binding to host cells and/or viral particles, and its nuclear localization strengthens the idea that lactoferrin is an important brick in the mucosal wall, effective against viral attacks and it could be usefully applied as novel strategy for treatment of viral infections.

## 1. Introduction

**Figure 1:**
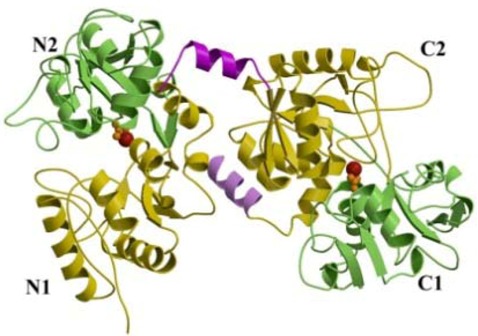
Structure of lactoferrin. From Baker and Baker [9].

Lactoferrin was identified in 1939 in bovine milk [1] and isolated in 1960 from both human [2,3] and bovine milk [4]. Lactoferrin, highly conserved among human, bovine, mouse, and porcine species, is a glycoprotein of about 690 amino acid residues belonging to the transferrin family, able to reversibly chelate two Fe(III) per molecule with high affinity (*K*d ~ 10^−20^ M) retaining ferric iron to pH values as low as 3.0, whereas transferrin retains iron to pH of about 5.5 [5,6]. The iron-binding affinity is high enough that, in the presence of lactoferrin or transferrin, the concentration of free iron in body fluids cannot exceed 10–18 M, thus preventing the precipitation of this metal as insoluble hydroxides, inhibiting microbial growth and hindering formation of reactive oxygen species. As is apparent from three dimensional (3D) structure of human lactoferrin (hLf) [7,8], the molecule is folded into two homologous lobes (N-lobe residues 1–333 and C-lobe residues 345–691). The two lobes are connected by a peptide (residues 334–344), which forms a 3-turn α-helix, whereas the peptide in transferrin is irregular and flexible. There are non-covalent interactions, mostly hydrophobic, where the two lobes pack together (Figure 1).

The amino acid sequence of hLf [10] has a high degree of identity with human transferrin (~60%), and the characteristic twofold internal sequence repeat suggests an ancestral gene duplication. The N and C-terminal halves have ~40% sequence identity.

## 2. Structure

### 2.1. Iron-Binding Sites

**Figure 2:**
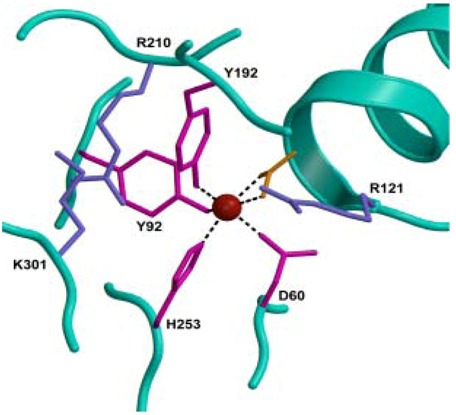
Iron binding site in the N-lobe of lactoferrin. From Baker and Baker [9].

The two lobes of lactoferrin are further divided into two domains (N1 and N2, C1 and C2) and each lobe binds one Fe(III) ion in a deep cleft between two domains (Figure 1). The iron sites are highly conserved in all iron-binding proteins, suggesting a common evolutionary origin [11,12]. The ligands for Fe(III) are the same in both lobes: One aspartic acid, two tyrosines, and one histidine (Asp-60, Tyr-92, Tyr-192, and His-253 in the N-lobe and Asp-395, Tyr-433, Tyr-526, and His-595 in the C-lobe), together with two oxygens from the CO_3_^2−^ anion (Figure 2).

Spectroscopic studies and the 3D structure suggest that the CO_3_^2−^ ion binds first, thus neutralizing the positive charge of the arginine residue (Arg-121 in the N-lobe and Arg-465 in the C-lobe) [6,13]. The participation of the CO_3_^2−^ ion in the iron coordination binding appears to be ideal for iron reversible binding [6] since the protonation of CO_3_^2−^ ion is a likely first step in the breakup of the iron site at low pH [14].

### 2.2. Conformational Changes

Iron binding and release are associated with large conformational changes in which lactoferrin adopts either an open or closed state. The iron-saturated form is closed and much more compact than the apo form [15]. In apo-lactoferrin, the N-lobe is in an open state, while the C-lobe is still closed, thus providing an important clue to the dynamic behaviour of the apo-protein. However, other crystal structures of lactoferrin of different species show some diversity in C-lobe that can adopt open forms, through the same kind of conformational change as was seen for the N-lobe [13].

The comparisons of structural and functional data on lactoferrin and transferrin have suggested the importance of cooperative interactions between the two lobes of the molecule, mediated by the α-helices. Both lactoferrin and transferrin share the property that their bound Fe(III) is spontaneously released *in vitro* at low pH. For transferrin, the iron release at low pH is considered to be important for iron delivery to cells [16]. The ferri-transferrin is internalized by transferrin receptor-mediated endocytosis, and then iron is released and the receptor is recycled to the cell surface.

Although there are indications that the transferrin receptor plays an active part in this process, the ability of transferrin to begin releasing iron at the endosomal pH of about 5.5 is also a critical factor. In contrast, lactoferrin retains Fe(III) to much lower pH, approximately 3.0. The key difference between lactoferrin and transferrin appears to be a cooperative interaction between the two lobes in lactoferrin that does not occur in transferrin. Iron release from the isolated N-lobe of lactoferrin begins at pH 5.0 [17], similarly to transferrin (pH 5.5). It can be supposed that in the absence of the lactoferrin C-lobe, Fe(III)-binding is substantially destabilized. Furthermore, studies on mutant lactoferrin have shown that when Fe(III)-binding in the N-lobe is disabled, Fe(III)-binding in the C-lobe is unaffected, while, when binding in the C-lobe is disabled, Fe(III)-binding in the N-lobe is destabilized, occurring at pH ~5.0 [18]. Therefore, there are cooperative interactions between the two lobes of lactoferrin through which Fe(III)-binding in the C-lobe stabilizes Fe(III)-binding in the N-lobe. Conversely, isolated N lobe of transferrin has an iron release at a pH identical to that of the intact protein. Therefore, the lactoferrin structure suggests that the C-terminal helix, which contacts the N-lobe close to the hinge, plays a very important role [19].

As in all lactoferrins, the linker peptide between the two lobes forms an α-helix, whereas in all transferrins has a flexible, extended and irregular structure. It can be hypothesized that the rigidity of the helical linker in lactoferrins allows a stronger interaction between the two lobes that stabilizes Fe(III)-binding in the N-lobe delaying the iron release at low pH [14].

### 2.3. Binding of other Metals

Lactoferrin is classified as an iron binding protein, but can also bind other metal ions including Cu^2+^, Mn^2+^, Zn^2+^, even if with lower affinity. Metal binding can be assayed by an increase in adsorption at 240–280 nm as consequence of ionization of the tyrosine ligands which bind to the metal ions [13]. The crystal structures of lactoferrin saturated with Mn^2+^ or Zn^2+^ have all shown closed forms, thus suggesting that lactoferrin could possess a role in binding other metal ions [9]. Moreover, it has been demonstrated that Mn^2+^- or Zn^2+^- saturated forms maintain some physiological functions of lactoferrin, unrelated to its iron binding capability [20] but probably related to its three remarkable concentrations of positive charge: Residues 1–7, 13–30 and inter-lobe region, close to the connecting helix [9].

### 2.4. Glycosylation

Lactoferrin is a glycosylated protein, possessing different number and location of putative glycosylation sites, according to different species [9]. In particular, hLf possesses three glycosylation sites (Asn-137, Asn-478 and Asn-623) and bovine lactoferrin (bLf) five (Asn-233, Asn-368, Asn-476 and Asn-545). The nature and the location of the glycosylation sites do not influence the polypeptide folding or iron and other molecules binding properties. Conversely, the loss of carbohydrate or sialic acid increases its sensitivity to proteolysis [9] or influences some physiological functions [20].

## 3. Human and Bovine Lactoferrin Gene Structure and Regulation

hLf gene maps to human chromosome 3p21.3 [21], while bLf gene is localized to chromosome 22 and syntenic group U12 [22]. The lactoferrin gene is organized into 17 exons. The size of the gene varies from 23 to 35 kb among human [23,24,25] and bovine species [26]. The signal peptide of lactoferrin consists of 19 amino acids, 11 of which are conserved within these two species. The first five amino acids of bovine protein include two basic amino acids, whereas the human protein begins at glycine and follows with four arginines, which make the hLf unique. The numbers of amino acids encoded by 15 of the 17 exons in these species are identical, and in 12 locations they have identical codon interruptions at the intron-exon splice junctions. Comparing the lactoferrin gene promoters from different species, common and different characteristics are observed. The hLf and bLf promoters contain a non-canonical TATA box, but only hLf has multiple steroid hormone response elements, while none are found in the other species studied, suggesting that the lactoferrin gene is differentially regulated among different species by steroid hormones [27]. The hLf gene expression is upregulated by estrogen with a magnitude of response that is cell-type specific (mammary glands, uterus) and by retinoic acids.

## 4. Concentrations in Human Body

Lactoferrin is expressed and secreted by glandular epithelial cells and by neutrophils. The highest levels (~7 g/L) is found in human colostrum [28], while it is also present at lower levels in mature milk, in most exocrine secretions (Table 1), and in the secondary granules of mature neutrophils [29,30]. Lactoferrin concentration increases in infection and/or inflammation sites due to the recruitment of neutrophils. 10^6^ neutrophils synthesize 15 μg of lactoferrin.

**Table 1:** Lactoferrin concentrations in human secretions.

Biological fluids	Concentration (mg/mL)
Colostrum	8
Milk	1.5–4
Tears	2
Saliva	0.008
Joint fluid	0.001
Vaginal secretion	0.008
Seminal fluid	0.112
Cerebrospinal fluid	Undetectable
Plasma	0.0004

## 5. Lactoferrin as Human Innate Defence against Infections

Even if lactoferrin and transferrin are similar in many respects, they possess different functions: Transferrin seems to exert a pivotal role in iron uptake by cells, whereas lactoferrin, which is found in many mucosal secretions, can be considered an important brick in the mucosal wall exerting a potent protective function.

**Figure 3:**
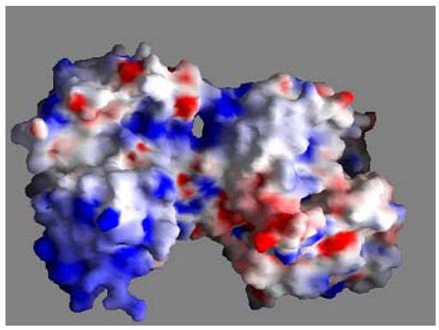
Distribution of surface charge of human lactoferrin. Blue: positive; red: negative. From Baker and Baker [9].

Unlike transferrin, the capability of lactoferrin to retain iron at acid pH, which characterizes infection and inflammation sites, together with its cationic nature (pI ~ 9) may be responsible for its ability to bind to various microbial and viral negative surface structures [31,32,33], and to anionic molecules such as DNA [34], heparin [35], glycosaminoglycans [36] could explain the different functions ascribed to this protein (Figure 3).

In mucosal secretions, which first have been injured by microorganisms, iron limitation (10–18 M) is considered in the healthy humans a physiological status hindering microbial growth. Conversely, an increase of iron concentration in the secretions, as a consequence of some pathologies, favours microbial virulence [37]. In human mucosa, peptides and proteins, including lactoferrin, symbolize the bricks of natural non-immune defences against microbial infections [38].

## 6. Antibacterial Activity of Lactoferrin Related and Unrelated to Its Iron Withholding Ability

The first function attributed to lactoferrin was antibacterial activity depending on its ability to sequester iron necessary for bacterial survival and growth [39]. This action of lactoferrin was considered bacteriostatic, as reversible by the addition of ferric iron [40].

However, bacterial pathogens are able to overcome iron limitation by means of two principal systems. The first is represented by the synthesis of small chelators, siderophores, which bind ferric iron with high affinity and transport it into bacteria through a specific receptor [41,42]. In addition to the synthesis of siderophores, some highly host-adapted bacterial species acquire iron directly through surface receptors able to specifically bind lactoferrin, and transport it across the outer membrane. The iron is bound by a periplasmic iron-binding protein, FbpA, and transported into the cell *via* an inner membrane complex comprised of FbpB and FbpC [43].

A lactoferrin bactericidal iron independent effect was also described [44]. A direct interaction between lactoferrin and lipopolysaccharide (LPS) of Gram-negative or lipoteichoic acid of Gram positive bacteria is required for the lethal effect [45,46,47]. Furthermore, it has been demonstrated that lactoferrin binds to the lipid A of LPS [48,49], inducing a release of LPS. This bactericidal activity of lactoferrin appears to be located in the N-terminal region as its derivative cationic peptide, called lactoferricin (Lfcin), is several fold more active than the intact protein [50,51,52]. However, the release of LPS can be annulled by high calcium concentration in the culture media [53]. As lactoferrin is also able to bind Ca(II) through the carboxylate groups of the sialic acid residues, present on two glycan chains, it cannot be ruled out that the release of LPS from Gram-negative bacteria can be also due to this additional binding property of lactoferrin [53].

## 7. Inhibition of Viral Infections by Lactoferrin

The antiviral activity of hLf was first demonstrated in mice infected with the polycythemia inducing strain of the Friend virus complex (FVC-P) [54]. Since 1994, a potent antiviral activity of both hLf and bLf against enveloped and naked viruses has been shown [55]. In most of these studies, when lactoferrin was tested both in apo- and in metal-saturated forms, no striking differences in the antiviral effect between the different forms were reported. Both lactoferrins act in the early phase of the viral infection thus preventing entry of virus into the host cell, either by blocking cellular receptors or by direct binding to virus particles [20]. bLf is often reported to exhibit higher antiviral activity than hLf [56].

Concerning lactoferricin, a pepsin-digested lactoferrin derivative, the antiviral activity of this highly positively charged loop domain of lactoferrin, was demonstrated for the first time by Andersen and co-workers [57] against human cytomegalovirus (HCMV) infection *in vitro*.

### 7.1. Herpesvirus

The *in vitro* activity of hLf and bLf against human cytomegalovirus (HCMV) infection has been described in 1994 [55]. Successively, other studies showed that both lactoferrin and cyclic lactoferricin prevented HCMV entrance into the host cells [58]. It has been reported that when negatively charged groups were added to lactoferrin by succinylation, the antiviral potency was mostly decreased, whereas the addition of positive charges through amination of the protein resulted in an increased anti-HCMV activity [57].

Successively other authors confirmed that lactoferrin inhibit the early steps of cytomegalovirus infection and that the antiviral effect is due to its cationic properties [59]. hLf as well as bLf, independently of iron-saturation or the presence of sialic acid, inhibited infection and replication of HSV-1 in human embryo lung cells [55]. Both hLf and bLf were found to prevent HSV-1 and HSV-2 cytopathic effect and yield in Vero cells [60,61].

The effectiveness of apo-lactoferrin on HSV-1 and HSV-2 infection was compared with that of metal ion saturated forms [Fe(III)-, Mn(II)-, Zn(II)-lactoferrin] and results of this study showed that the antiviral effect of the differently saturated bLf towards both viruses was mainly exerted during the initial viral adsorption phase [61].

In the attempt to identify the regions of lactoferrin responsible for the anti-HSV-1 activity, the inhibiting effect of peptide fragments, derived from the tryptic digestion of bLf, were also analyzed [62]. Among high molecular weight peptides, one fragment corresponding to the C-lobe was ten-fold more effective than another one corresponding to a large portion of the N-lobe. On the other hand, this last one was still six-fold less active than native bLf [62,63]. Two negatively charged small peptides deriving from N-lobe, previously shown effective on HSV-1 infection, have been further studied and results of this research demonstrated that the net negative charge of these peptides was not responsible for the antiviral activity [64].

It is well known that the initial attachment of HSV to cells occurs through binding of the viral glycoprotein(s) gC or gB to heparan sulfate of host cells. In the absence of HS, virus can bind to chondroitin sulfate proteoglycans, although with lower efficiency [65]. Marchetti and co-workers [66] demonstrated that bLf was a strong inhibitor of HSV-1 infection in cells expressing either heparan sulfate or chondroitin sulfate or both, but was ineffective or less efficient in glycosaminoglycan deficient cells or in cells treated with glycosaminoglycan-degrading enzymes, suggesting that the anti-HSV-1 activity of lactoferrin is dependent on its interaction with cell surface glycosaminoglycan chains of heparan sulfate and chondroitin sulfate [65].

The mechanism of inhibiting activity of both hLf and bLf against HSV-2 has been further investigated [67]. The antiviral effect of these proteins towards HSV-2 strain and its glycoprotein C (gC)-truncated derivative HSV-2 gC-neg1 has been tested in monkey kidney cells. The results indicated that the antiviral activity of bLf does not involve gCeHS interaction as there was no difference in its effectiveness towards wild type and mutant virus. As regards hLf, the mutant virus HSV-2 gC-neg1 was more sensitive compared with the wild type, suggesting that the human protein might interact with some viral structures that in wild-type viruses are masked by gC. When the modulation of HSV-2 infection by bLf and hLf was investigated under different experimental conditions, the bovine protein proved more effective than the human protein. Moreover, differently from what observed with HSV-1, bLf inhibited HSV-2 plaque-forming activity also in cells devoid of GAG expression, thus suggesting that bLf may block a virus receptor of non-GAG nature [67]. This observation adds new information on the anti herpes virus activity of this protein, confirming it as an outstanding candidate for the treatment of herpetic infections.

Concerning hLf, in addition to inhibiting the adsorption and post-attachment events of HSV-1 infection, hLf is also able to neutralize HSV-1 and that the inhibition of cell-to-cell spread involves viral gD [68].

Andersen and co-workers [69] demonstrated that bovine Lfcin inhibited HSV-1 and HSV-2 infection probably by blocking the entry of the virus and that the human homolog (amino acids 18–42), which shares 36% sequence similarity with Lfcin (amino acids 17–41), displayed much lower antiviral activity. The same authors [70] demonstrated that also Lfcin was dependent on the presence of heparan sulfate at the cell surface to exert its antiviral activity. Other studies demonstrated that lactoferrin and several of the Lfcin derivatives exhibited similar affinity for heparan sulfate, but the lactoferrin proteins were more active compared with the smaller peptides [71].

The antiviral activity has also been reported for human Lfcin [72]. The anti-HSV activity of Lfcin seems to involve viral interaction with the cell surface glycosaminoglycan heparan sulfate, thereby blocking viral entry. Lfcin inhibited cell-to-cell spread of both HSV-1 and HSV-2. Inhibition of cell to-cell spread by bovine Lfcin involved cell surface chondroitin sulfate. Based on transmission electron microscopy studies, human Lfcin, like bovine Lfcin, was randomly distributed intracellularly, thus differences in their antiviral activity could not be explained by differences in their distribution. In contrast, the cellular localization of iron-saturated (holo)-lactoferrin appeared to differ from that of apo-lactoferrin, indicating that holo- and apo- lactoferrin may exhibit different antiviral mechanisms [72].

Lactoferrin and Lfcin agaist HSV-1 cellular uptake and intracellular trafficking were further studied by immunofluorescence microscopy. In comparison to the untreated infected control cells, both the bLf- and bovine Lfcin-treated cells showed a significant reduction in HSV-1 cellular uptake. The few virus particles that were internalized appeared to have a delayed intracellular trafficking. Thus, in addition to their interference with the uptake of the virus into host cells, lactoferrin and Lfcin also exert their antiviral effect intracellularly [73,74].

Concerning *in vivo* studies against CMV, experiments in BALB/c mice showed that the administration of bLf, before murine CMV infection, completely protected mice from death [75]. Successively, other authors also analyzed the anti-cytomegalovirus activity of lactoferrin *in vivo* in rat models with and without immune suppression, demonstrating that treatment with lactoferrin (intravenously) was helpful when infection was initiated with cell-free virus, but not with virus infected leukocytes and that lactoferrin exerted its effects *via* inhibition of cell entry rather than *via* stimulation of the immune system [59]. In *in vivo* studies on HSV-1, it has been demonstrated that topical administration of 1% bLf, prior to the virus inoculation, suppressed HSV-1 infection in the mouse cornea but not viral propagation [76]. The influence of bLf feeding on the HSV-1 cutaneous infection of mice has been evaluated and results of this study, in which mice were infected with HSV-1 ten days after lactoferrin administration, showed that lactoferrin inhibited the appearance of skin lesions [77].

Recently, it has been also reported that cervicovaginal lavage differently inhibited HSV infection by a mean value of approximately 57% during the follicular or luteal phase, but only by 36% in hormonal contraceptive users [78]. Being lactoferrin synthesis under steroid control, its influence on the antiviral activity of cervical fluids cannot be ruled out.

Concerning animal herpes virus, it has been reported that exposure of susceptible cells to bLf prior or during viral adsorption strongly inhibited feline herpes virus 1 (FHV-1) replication [79]. Other studies demonstrated that both the apo- and holo-lactoferrin inhibited canine herpes virus multiplication in Madin-Darby canine kidney (MDCK) cells [80].

### 7.2. Human Immunodeficiency Virus (HIV)

It has been demonstrated that both bLf and hLf were able to inhibit the HIV-1-induced cytopathic effect. Addition of negatively charged groups to lactoferrin by succinylation resulted in a strong antiviral effect on HIV-1 and HIV-2, while the addition of positive charges to lactoferrin through amination resulted in a loss of anti-HIV activity [58,81,82]. Both HIV-1 replication and syncytium formation were efficiently inhibited, in a dose-dependent manner, by apo- or holo-, Mn(II) and Zn(II)- lactoferrin [83]. Other studies demonstrated that bLf strongly inhibited viral reverse transcriptase but only slightly inhibited HIV-1 protease and integrase [84]. Studies on bLf-resistant HIV-1 variants showed that the viral envelope protein, which contains two mutations that are associated with an altered virus-host interaction and a modified receptor-co-receptor interaction, mediated the bLf resistance phenotype demonstrating that bLf targeted the HIV-1 entry process [85]. Recently, when proteins from milk and serum were tested for their ability to block dendritic cell-mediated HIV-1 transmission, bLf turned out to be the most potent inhibitor [86]. Finally, a synergy between lactoferrin in combination with Zidovudine against HIV-1 replication *in vitro*, has been reported [87].

It has been reported that oral administration of bLf suppressed oral inflammation in feline immunodeficiency virus FIV-infected cats with intractable stomatitis infection. This result suggests that bLf therapy may have a potential application to improve and protect functions of overactivated lymphocytes by modulating the cell proliferation, cell cycle and cytokines expression as shown in cats in terminal stage of FIV infection [88].

### 7.3. Friend Virus Complex (FVC)

The effects of lactoferrin treatment on the development of erythroleukemia in the spleen of mice infected with FVC were studied [89]. The treatment was started at days 7 and 14 before viral infection and days 0, 1, 3, 7, and 11 after viral infection, and in the spleens were analyzed 14 days after infection. In mice whose treatment was initiated at days 0 and 1 few leukemic cells were present in the spleen whereas in mice whose treatment was initiated at day 3 leukemic cells began to spread out in the red pulp and encroached upon the white pulp and in mice whose treatment was initiated at days 7 and 11 many leukemic cells were present in the red pulp. The morphologic features of the spleen in animals, whose treatment was initiated at day 7 or 14 before viral infection, were similar to those of untreated control groups [89]. Results of another *in vivo* study suggested that the protective effect of holo lactoferrin was probably due to an action on cells responding to the FVC or to an action on cells which influence the cells responding to the FVC or which influence the virus [54]. Finally, holo-lactoferrin and recombinant murine (rmu) interferon γ (IFNγ), alone or in combination, were used to influence disease progression in mice infected with the polycythemia-inducing strain of the Friend virus complex (FVC-P). Results of this study showed that spleen focus forming virus (SFFV) titers and levels of SFFV mRNA and genomic DNA dramatically decreased in mice treated with the combination of lactoferrin and rmu-IFNγ. Moreover, the combined treatment also enhanced the survival rates of FVC P-infected mice, suggesting a synergistic suppressive effect of lactoferrin with rmu-IFNγ on disease progression in FVC-P-infected mice [90].

### 7.4. Human Hepatitis C Virus (HCV)

Both bLf and hLf effectively prevented human hepatitis C virus (HCV) infection in cultured human hepatocytes (PH5CH8), bLf being the most active. In this study, a direct interaction between lactoferrins and E1 and E2 HCV envelope proteins has been reported [91]. It has been also demonstrated that pre-incubation of HCV with bLf inhibits viral infection, while cell pretreatment with bLf was ineffective [92].

Further studies demonstrated that bLf inhibited HCV entry into the cells by interacting with viral particles immediately after mixing of bLf and HCV inoculum [93]. Nozaki and co-workers [94] better characterized the binding activity of lactoferrin to hepatitis C virus E2 envelope protein and determined the region of lactoferrin important for this activity. Results from this study provided the first identification of a natural protein-derived peptide that, specifically binding HCV E2 protein, prevented HCV infection [94].

Successively, 33 amino acid residues (termed C-s3-33; amino acid 600-632) from hLf were found to be primarily responsible for the binding activity to the HCV E2 envelope protein and for the inhibiting activity against HCV infection. If this sequence was repeated two or three times, two or three C-s3-33 repeated sequences possessed a stronger antiviral activity than of C-s3-33, thus suggesting that tandem repeats of lactoferrin-derived anti-HCV peptide are useful as anti-HCV reagents [95]. Other two helical peptides deriving from lactoferrin were found to bind hepatitis C virus envelope protein E2 [96].

Concerning *in vivo* studies, following the first pilot study of Tanaka and co-workers [97], a trial was designed to evaluate the relationship between the dose of bLf and its effect on serum alanine aminotransaminase and HCV RNA levels in forty-five patients with chronic hepatitis C [98]. The excellent tolerance and potential anti-HCV activity of bLf shown in this trial suggested that further trials using a large number of patients were obligatory. In a successive study the effects of long-term oral administration of bLf on serum parameters in patients with chronic hepatitis C have been analyzed and results obtained suggested that oral administration of lactoferrin induced a Th1-cytokine dominant environment in the peripheral blood so favouring the eradication of HCV by a combined interferon therapy [99]. It has also been demonstrated that an elevated percentage of HCV infected patients were endotoxemic [100]. These patients were poor responders to the IFNα/ribavirin treatment and exhibited high serum levels of lactoferrin antibodies that affected the antiviral activity of lactoferrin and abrogated the lactoferrin binding to lipopolysaccharides. This interaction inhibited the binding of lipopolysaccharide to lipopolysaccharide-binding protein, thus preventing its fixation to CD14 (+) cells and leading to a reduced release of pro-inflammatory cytokines [101].

Concerning the effectiveness of oral bLf mono therapy, in a clinical trial patients with chronic hepatitis C randomly received either oral bLf at a dose of 1.8 g daily for 12 weeks, or an oral placebo. There was no significant difference in viral response rates between the two groups, indicating any significant bLf efficacy in patients with chronic hepatitis C [102].

Different results have been obtained by comparing the viral response to bLf mono therapy at higher doses (daily dose of 3.6 g instead of 1.8 g) for 8 weeks followed by bLf, interferon and ribavirin combined therapy for 24 weeks. The results showed that the decrease in HCV RNA titer by lactoferrin mono therapy contributes to the effectiveness of the combined therapy of interferon and ribavirin in patients with chronic hepatitis C [103].

An interesting observational study has been reported on Egyptian patients feed with camel milk which contains lactoferrin. In *in vitro* model, purified camel lactoferrin interacts with HCV, thus leading to a complete virus entry inhibition [104].

### 7.5. Human Hepatitis B Virus (HBV)

Lactoferrin also prevents HBV infection in cultured cells and, differently to HCV, cell pretreatment with lactoferrin was required to inhibit HBV infection. As pre-incubation of HBV with bLf had no inhibitory effect on viral infection, these results suggested that bLf interaction with susceptible cells was important for its anti-HBV effect [105]. However, it was unclear whether bLf could inhibit HBV amplification in HBV-infected cells.

Recently, Li *et al.* reported that bLf, and its iron-, and zinc-saturated forms significantly inhibited the amplification of HBV-DNA in a dose-dependent manner in HBV-infected HepG2 cells, while bLf hydrolysate were ineffective [106].

Mother-to-child transmission of HBV is among the most important causes of chronic HBV infection and is the commonest mode of transmission worldwide. WHO postulates that chronic HBV infection of the mother could not be an argument against breastfeeding. Even if breast milk provides a number of bioactive including lactoferrin, there have not been sufficient studies that can even partially explain the possible effect of breastfeeding on eventual prevention of mother-to-child transmission of HBV [107].

### 7.6. Respiratory Syncytial Virus (RSV) and Parainfluenza Virus (PIV)

It has been demonstrated that lactoferrin inhibited both RSV absorption and growth *in vitro* nevertheless its antiviral activity was low when added to an infant formula [108]. Successively, Sanoand co-workers [109] showed that RSV-induced IL-8 secretion from HEp-2 cells was down regulated by lactoferrin and that both RSV infectivity and uptake were decreased by lactoferrin treatment. Toclarify the mechanism of this effect, the interaction of lactoferrin with RSV F protein, the mostimportant surface glycoprotein for viral penetration, was examined and results obtained showed thatlactoferrin directly interacted with the F (1) subunit, which involved antigenic sites of F protein [109].Concerning PIV, Lf exhibits antiviral activity against hPIV-2 by inhibiting virus adsorption to the surface of the cells thus preventing viral infection and replication [110].

### 7.7. Alphavirus

The mechanism of hLf antiviral activity was also investigated by utilizing alphaviruses (Sindbis virus and Semliki Forest virus) adapted or non-adapted to interaction with heparan sulfate [111]. Results obtained demonstrated that lactoferrin was able to prevent *in vitro* infection only by heparin sulfate-adapted viral strains suggesting that hLf inhibited infection of heparan sulfate-adapted alphavirus by interfering with virus-receptor interaction [111].

### 7.8. Hantavirus

Hantaviral foci number, in cultured cells infected with SR-11, was reduced with bLf treatment [112]. Mechanisms of anti-hantaviral activities of bLf and ribavirin (Rbv) were also investigated. Preincubation of cells with bLf before infection inhibited hantavirus focus formation of 85% whereas post infection treatment with Rbv inhibited the focus formation of 97.5%. Conversely, other *in vitro* experiments showed that Hantaan hantavirus, the prototype hantavirus, is insensitive to several antiviral salivary proteins, and is partly resistant to the antiviral effect of saliva [113].

It has been found that combined bLf and Rbv treatment completely prevented focus formation [114]. Consequently, in *in vivo* studies, bLf pre- and Rbv post-treatment were evaluated in suckling mice infected with hantavirus, of which 7% survived. Lactoferrin administered before viral challenge improved survival rates to up to 70% for single administration and up to 94% for double administration. Rbv gave survival rates up to 81%. These results suggested that both lactoferrin and Rbv were efficacious in the treatment of hantavirus infection *in vivo* [114].

### 7.9. Human Papillomavirus (HPV)

Results of studies carried out utilizing HPV 16-like particles and cultured cells demonstrated that lactoferrin acted early in the HPV uptake process with a dose-dependent relationship and that bLf was a more potent inhibitor of HPV entry than hLf [115]. Differently, bLf and hLf were found both potent inhibitors of HPV-5 and -16 infections [ex115ora 116]. Moreover, bovine Lfcin 17–42 and human Lfcin 1–49 had an antiviral effect and this efficacy differed depending on size, charge and structures of the Lfcin [116,117].

### 7.10. Rotavirus

The anti-rotavirus effect of bLf was tested in cultured human intestinal cells (HT-29 cells), expressing the differentiation phenotype of mature enterocytes, the *in vivo* target of rotavirus infection [118]. Results obtained showed that bLf prevented either virus attachment to intestinal cell receptors or an unknown post adsorption step. Although the antiviral activity was mediated by the N-lobe [119], it was hypothesized that bLf prevention of viral attachment was not related to a competition for common binding sites on HT-29 cells, since the rotavirus strain utilized in this study binds to glycidic residues different from glycosaminoglycans [120], and flow cytometry assays demonstrated a specific interaction of lactoferrin with viral particles. The bLf inhibition in the post adsorption step could be attributed to the withholding of calcium, which is important for the morphogenesis of the virus. Other studies investigated the role of metal binding, sialic acid and tryptic fragments of bLf in the activity towards rotavirus infection [119], Results obtained demonstrated that the effect of differently metal saturated lactoferrin was exerted during and after the viral attachment step, the removal of sialic acid enhanced the anti-rotavirus activity of lactoferrin, and that a large fragment (86–258) and a small peptide (324–329: YLTTLK) obtained by tryptic digestion of bLf were able to inhibit rotavirus even if at lower extent than undigested protein [119].

The effect of whey protein concentrate supplemented with or without lactoferrin on a rotavirus infection model in suckling rats has been also investigated, focusing on the diarrhoea process and gut and systemic host immune function. Whey protein concentrate supplemented with or without lactoferrin reduces the severity of rotavirus-induced acute gastroenteritis and modulates the immune response against the pathogen [121].

### 7.11. Feline Calicivirus (FCV)

Incubation of bLf cultured cells either before or together with FCV inoculation substantially reduced FCV infection. Lactoferrin was detected on the surface of cells by immunofluorescence, suggesting that the interference of viral infection may be attributed to lactoferrin binding to susceptible cells, thereby preventing the attachment of the virus particles [122]. Lfcin also reduced FCV infection [122]. It has been further found that increasing the net negative charges of lactoferrin by acylation eliminated its antiviral effects against feline calicivirus [123].

### 7.12. Adenovirus

Both bLf and hLf prevented adenovirus infection *in vitro* in a dose-dependent manner [124] and, as already reported for other viruses, bLf showed the highest antiviral activity. Differently from that observed with poliovirus [125] and in agreement with data reported for many other virus models, metal-saturation of bLf did not significantly influence its activity against adenovirus infection. bLf inhibited the early step of viral infection, preventing adenovirus antigen synthesis only when pre incubated with epithelial cells or when added during the attachment step. bLf activity was mediated by the N-lobe, whereas the C-lobe lacked of any effect [126]. Moreover, bovine Lfcin is able to prevent adenovirus infection [127].

This antiviral activity of bLf occurs through bLf interaction with adenovirus particles and in particular, with the adenovirus penton base (polypeptide III), the protein responsible for viral attachment to the integrin cell receptors [128].

However, the interaction of bLf with host cell receptors cannot be excluded. The primary receptor described for infection of most adenovirus species (A, C, D, E and F) was the coxsakievirus-adenovirus receptor (CAR) [129,130,131]. The infection of adenovirus serotype 5 (species C), a common human pathogen exploited as a viral vector for gene therapy and vaccination, involves binding of the viral fiber knob to CAR on the target cells [132], followed by an interaction between the viral penton base with integrins on the cell surface [133]. It has been reported that adenoviral infection was prominently enhanced by bLf but not hLf, and was not prominently enhanced using blood monocyte-derived macrophages, suggesting that the relevant receptor is expressed on monocyte-derived dendritic cells [134]. These data are conflicting with those showing an antiviral effect against adenovirus by both hLf and bLf [124,126].

Concerning adenovirus serotypes 19 and 37 shown to be etiological agent of epidemic keratoconjunctivitis [135], it is important to underline that the tears contain lactoferrin, produced in the acinar cells of the lacrimal gland, at a concentration of around 2.2 mg/mL [136]. In tears, due to the high concentration of free lactoferrin, it is most likely that lactoferrin provides a protective role against viral adhesion and pathogenesis [137]. Conversely, commercial hLf (Sigma-Aldrich) was found to promote adenoviral infection of corneal epithelial cells [138] as well as hLf or bLf from milk or recombinant hLf from rice to enhance adenoviral infection of some myeloid cells [139] This paradoxical enhancement of adenoviral infection by lactoferrin should be explained by the high degree of degradation of commercial hLf showed by Johansson *et al.* [138], and by different glycosilation sites of rhLf, as well as by the degrees of purity or of iron saturation not reported by Adams *et al.* [139].

### 7.13. Picornavirus

Studies on poliovirus type 1 infection in Vero cells demonstrated that both bLf and hLf inhibited viral cytopathic effect with a dose-dependent relationship by interfering with an early step of viral infection [125]. Other researchers have studied the ability of bLf fully saturated with ferric, zinc and manganese ions to prevent poliovirus infection. Results obtained demonstrate that only Zn(II)-lactoferrin was capable of inhibiting infection when added after the viral adsorption step. As the inhibition was proportional to the different degrees of lactoferrin metal saturation, the possibility that this phenomenon could be mediated by the intracellular delivery of metal ions, already demonstrated for iron saturated lactoferrin [140], has been investigated. This hypothesis was confirmed by the dose34 dependent inhibition obtained with the addition of different zinc sulfate concentrations to infected monolayers [125]. It is likely that zinc could interfere with viral protein maturation as it has been previously demonstrated that the inclusion of zinc, at a concentration that inhibits the proteolytic post translational processing of poliovirus polyprotein, resulted in impaired poliovirus infection [141]. Moreover, the incubation of poliovirus in a Zn(II) containing buffer resulted in marked structural alterations of the capsid and an increase in the permeability to RNase, so that the infectivity of the virus was significantly affected [142].

hLf and bLf were also assayed *in vitro* to assess their inhibiting capacity on the cytopathic effect of enterovirus 71 (EV71) on human embryonic rhabdomyosarcoma cells [143]. Both proteins were found to be potent inhibitors of EV71 infection, with bLf being the most active. Results from kinetic experiments suggested that lactoferrin probably exerted its effect on viral adsorption.

An interesting study has been carried out in a transgenic mouse model for demonstrating the protective effects of recombinant lactoferrin against EV71 infection. Transgenic mice carrying alpha lactalbumin-porcine lactoferrin and BALB/c wild-type mice were infected with EV71. Following EV71 inoculation on the 4th day of life, pups ingesting transgenic milk showed the significantly higher survival rate and heavier body weight compared with wild-type mice. RT-PCR analysis for EV71 viral RNA showed that the recombinant porcine lactoferrin had a blocking effect on EV71 infection. Our data suggest that oral intake of porcine lactoferrin-enriched milk exhibited the ability to prevent EV71 infection [144].

The effect of lactoferrin on echovirus 6 infection *in vitro* was also investigated [145]. Results of this study showed that echovirus 6 infected cells die as a result of apoptosis and that programmed cell death is inhibited by bLf treatment. This was the first report in which the prevention of viral-induced apoptosis by lactoferrin was demonstrated [145].

The same authors have successively investigated the mechanism of bLf anti-echoviral effect demonstrating that echovirus infects susceptible cells by an endocytic pathway and that lactoferrin treatment is able to prevent viral genome delivery into the cytoplasm. It is likely that lactoferrin interaction with echovirus capsid proteins induces alterations that stabilize the conformation of the virion making it resistant to uncoating.

Taken together the results of these studies, the inhibition of echovirus 6 infectivity by lactoferrin seems to be dependent on its interaction not only with cell surface glycosaminoglycan chains but also with viral structural proteins, demonstrating that this glycoprotein targets the virus entry process [146]. On the other hand, the lactoferrin efficacy could be based on its positive charge, because the increasing of net negative charges by acylation eliminated antiviral effects [123].

### 7.14. Rhinovirus

Lactoferrin did not inhibit rhinoviruses, while human milk decreased the growth of some of the rhinoviruses [147].

### 7.15. Influenza A Virus

Influenza is one of the main plagues worldwide. The statistical likelihood of a new pandemic outbreak, together with the alarming emergence of influenza virus strains that are resistant to available antiviral medications, highlights the need for new antiviral drugs.

It has been found in *in vitro* model that cell cultures died as a result of apoptosis following to H3N2 influenza A virus infection. Similarly to that first demonstrated in echovirus 6, bLf treatment inhibited programmed cell death by interfering with function of caspase 3, a major virus induced apoptosis effector, as well as blocked nuclear export of viral ribonucleoproteins so preventing viral assembly [148].

Since 2003, H5N1 avian influenza A virus was detected and identified in South East Asia. Both the native and esterified bLf seem to be the most active antiviral proteins among the tested samples, followed by b-lactoglobulin. a-Lactalbumin had less antiviral activity even after esterification [149].

### 7.16. Japanese Encephalitis Virus

It has been hypothesized that bLf could be effective against japanese encephalitis virus (JEV) through its ability to bind to glycosaminoglycan, one possible receptor for JEV. The results showed that bLf inhibited the early events essential to initiate JEV infection, which includes blocking virus attachment to cell membranes and reducing viral penetration. Even if these results support the premise that the interaction of bLf with cell surface expressed glycosaminoglycans, plays an essential role in the antiviral activity, bLf was functional in inhibiting viral entry into heparin sulfate-deficient cells. This finding provided evidence to suggest that also cell surface-expressed low-density lipoprotein receptor LDLR may play a role in JEV infection [150].

### 7.17. Tomato Yellow Leaf Curl Virus

The antiviral activity of native and esterified whey protein fractions including lactoferrin was studied to inhibit tomato yellow leaf curl virus (TYLCV) on infected tomato plants. Whey proteins fractions and their esterified derivatives were sprayed into TYLCV-infected plants. Samples were collected from infected leaves before treatment, 7 and 15 days after treatment for DNA and molecular hybridization analysis. Native and esterified lactoferrin showed complete inhibition after 7 days [151].

## 8. Conclusions

The protective effect of lactoferrin towards microbial infections has been widely demonstrated in a large number of *in vitro* studies. Its high cationic feature favors the binding to microbial and viral surface components as well as to heparansulfate proteoglycans (HSPG), cell receptors for bacterial adhesion and enveloped viral particle early interactions. The capability of lactoferrin to exert antiviral activity, through its binding to host cells or viral particles or both, strengthens the idea that this glycoprotein is an important brick in the mucosal wall, effective against viral attacks. During viral infection, the epithelium can be injured, with the consequence of loss of integrity and protection. As a matter of fact, the mucosa plays an important role as a protective physical and functional barrier between the external environment and underlying tissues, while the components of its secretions, especially lactoferrin are central elements in the initiation and regulation of innate and adaptive immune responses.

It is believed that the magnitude of inflammation is a major contributing factor to viral disease severity [152]. Epidemiological evidence and clinical observations of natural infections in humans suggest that different viruses may be associated with different inflammatory responses. Whether or not these differences can be attributed to the viruses themselves or to hosts that are susceptible to severe infection or prone to produce high levels of inflammation with a given virus is unknown.

In this context it should be important to consider the role of lactoferrin in *in vivo* modulating the type or magnitude of the inflammatory response during viral infections. Unfortunately, few clinical trials on lactoferrin efficacy against viral infections have been carried out. The scarcity of clinical trials hinders to compare the inflammatory response and the lactoferrin efficacy in different animal models infected with different viruses.

However, the antiviral activity of lactoferrin detected in cultured cell monolayers infected by enveloped and naked viruses, has been found to be not related to the degrees of lactoferrin iron saturation, while Zn- and Mn-saturated lactoferrin exerted a potent antiviral capacity against HSV, HIV and poliovirus infection [61,83,125].

Conversely, lactoferrin antiviral activity is strongly related to its binding to viral particles or to host cells or both. Lactoferrin antiviral activity is also associated to the prevention of Echovirus 6- and H3N2 influenza virus-induced apoptosis [145,148].

**Figure 4:**
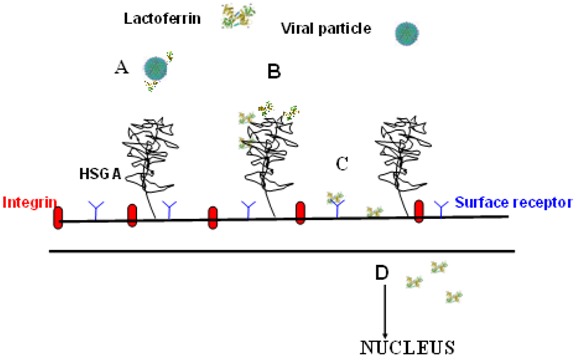
Different mechanisms of lactoferrin in preventing viral infection.

Figure 4 shows the different mechanisms of lactoferrin in preventing viral infection: The binding to viral particles (A), the binding to heparan sulfate glycosaminoglycans (HSGA) (B),the binding to viral receptors (C), and intracellular localization (D), involving apoptosis or inflammatory pathways.
